# Investigation of *Chlamydiaceae *in semen and cauda epididymidis and seroprevalence of *Chlamydophila abortus *in breeding bulls

**DOI:** 10.1186/1751-0147-52-2

**Published:** 2010-01-13

**Authors:** Ann-Charlotte Karlsson, Stefan Alenius, Camilla Björkman, Ylva Persson, Stina Englund

**Affiliations:** 1 Division of Ruminant Medicine and Veterinary Epidemiology, Department of Clinical Sciences, Faculty of Veterinary Medicine and Animal Science, Swedish University of Agricultural Sciences (SLU), Box 7054, SE-750 07, Uppsala, Sweden; 2 Department of Animal Health, Section for Farm Animal Health and Welfare, National Veterinary Institute, Pb 750 Sentrum, N-0106 Oslo, Norway; 3 Department of Animal Health and Antimicrobial Strategies, Section of Farm animals, National Veterinary Institute (SVA), SE-751 89, Uppsala, Sweden; 4 Department of Animal Health and Antimicrobial Strategies, Section of Antibiotics, National Veterinary Institute (SVA), SE-751 89, Uppsala, Sweden

## Abstract

**Background:**

Reproductive disorders associated with chlamydial infection have been reported worldwide in cattle and there are indications of potential venereal transmission.

**Methods:**

Semen samples from 21 dairy bulls and cauda epididymidis tissue samples from 43 beef bulls were analysed for chlamydial agent by real-time polymerase chain reaction (PCR) including an internal amplification control (mimic). Additionally, presence of antibodies against *Chlamydophila (Cp.) abortus *among the bulls was investigated with the commercial Pourquier^® ^ELISA *Cp. abortus *serum verification kit.

**Results:**

No chlamydial agent was detected by PCR in either the semen samples or in the tissue samples. Additionally, no antibodies against *Cp. abortus *were detected.

**Conclusions:**

The results suggest that *Cp. abortus *is very rare, or absent in Swedish bulls and thus the risk for venereal transmission of chlamydial infection through their semen is low. However, because *Chlamydophila *spp. infection rates seem to differ throughout the world, it is essential to clarify the relative importance of transmission of the infection through semen on cattle fertility.

## Background

Bovine chlamydiosis has been associated with several disease manifestations [1]. Reproductive disorders such as sporadic abortions and reduced fertility, linked with chlamydial infection have been reported from Germany [2,3], Great Britain [4], Italy [5], Japan [6], Switzerland [7], Taiwan [8] and the USA [9]. In Sweden, the incidence of abortion in cows is low. However, reproductive disorders and infertility are major causes of culling but are often difficult to be diagnosed. Chlamydial infection in bulls may be the cause to some of these problems [10]. Experimental studies have shown that the bacteria can be excreted in semen of inoculated bulls and rams [11] and isolation of the agent from semen of naturally infected bulls and rams has been reported [12-14]. The vaginal mucosa in sheep and uterine mucosa in cattle are susceptible to infection [15,16] and transmission of chlamydial agent by experimentally infected semen to heifers and sheep has been demonstrated [17,18].

The two species *Chlamydophila (Cp.) abortus *and *Cp. pecorum *are known to infect cattle and are suggested to be ubiquitous [9,19]. Moreover, *Cp. psittaci *infections in cattle have been reported [20,21]. All three species have been identified in bull semen [22,23]. *Cp. abortus *is the cause of Ovine Enzootic Abortion (OEA), the major infectious cause of abortion and lamb loss with great economic losses in many sheep-producing countries [24]. *Cp. pecorum *has foremost been associated with polyarthritis, encephalitis and inapparent intestinal infection, and the impact by *Cp. psittaci *in ruminants is yet to be investigated.

Each year about 80 top-ranked performance-tested yearling beef bulls are sold all over Sweden, mainly to pedigree breeders, after six months of testing at the only performance testing station in the country. These performance-tested bulls represent the best-documented beef bulls with the highest impact on the breeding programme in Sweden and are therefore important potential transmitters of *Chlamydophila *spp. by venereal route. Additionally, artificial insemination (AI) is performed yearly on more than 95% of the approximately 400,000 Swedish dairy cows [25]. As there is a possibility of transmission of *Chlamydophila *spp. via this route, it is important to determine whether breeding bulls are infected through screening of semen before AI in order to minimize this risk. The aim of this study was to investigate the presence of chlamydial agent in semen and in tissue of cauda epididymidis and to estimate the seroprevalence of *Cp. abortus *in Swedish bulls.

## Methods

### Animals and samples

#### Beef bulls

This study comprises samples from a subset of 166 beef bulls from 124 herds from different parts of Sweden that were taken to the only performance testing station in Sweden in September 2002. On arrival the bulls were approximately six months old. They were divided into groups, based on breed and body weight and placed in ten adjacent semi-outdoor pens under the same roof. The bulls were weighed every second week throughout the testing period (September-March) and at the end of the period, an individual growth index was calculated. Bulls with fast growth rates were sold at livestock auction and bulls with growth indexes below the threshold, stated by the breeders' organisations, were either slaughtered or returned to their owners. In total, 43 of the beef bulls that were sent to slaughter were included in this study (23 Charolaise, 7 Hereford, 6 Simmental, 3 Aberdeen Angus, 3 Limousine and 1 Blonde d'Aquitaine). The daily growth rates of these bulls were somewhat lower than the bulls sold at auction, but were still higher than the growth rates of non-tested beef sires in Sweden [26]. Because of co-operation with another study and their definite criteria [27], only bulls with clinically normal reproductive organs and scrotal circumference above 30 cm were included.

Testes and epididymides were removed at the time of slaughter and immediately put in a container with crushed ice and transported refrigerated to the laboratory where they arrived the next day. An incision (approx. 1.5 cm long and 0.5 cm deep) was made with a scalpel blade in the middle, distal part of the cauda epididymides [28], and a sample of 0.5 × 0.5 cm was taken and stored at -70°C until used for DNA preparation. In addition, blood samples for serum preparation were taken on arrival (6 month of age) and at departure (1 year of age) from the testing station. Sera were stored at -20°C.

#### Dairy bulls

Semen samples (0.2 ml payettes) and sera from 21 dairy bulls about 1 year old (Swedish Holstein and Swedish Red) in service, were submitted to the laboratory from one of the only two semen producing companies in Sweden, Svensk Avel http://www.vikinggenetics.com. Semen samples were stored at -70°C prior to preparation for analysis by real-time polymerase chain reaction (PCR). Sera for serology were stored at -20°C until analysed.

### Detection of *Chlamydiaceae *by real-time polymerase chain reaction

DNA was extracted from semen and cauda epididymidis samples for PCR analysis using a High Pure Template Preparation kit, following manufacturer's instructions (Roche Diagnostics, Basel, Switzerland) and stored at -20°C. Analyses were performed using a *Chlamydiaceae*-specific real-time PCR protocol developed by Everett and others [29], targeting the 23S ribosomal DNA. Briefly, the primers used were TQF (5'-GAA AAG AAC CCT TGT TAA GGG AG-3') and TQR (5'-CTT AAC TCC CTG GCT CAT CAT G-3'). The sequence of the fluorescent FAM-labelled probe was 5'-CAA AAG GCA CGC CGT CAA C-3'.

An internal amplification control (mimic) was constructed and used to detect false negative PCR results, as previously described [30]. The primers used in the mimic producing PCR were TQFActin (5'-GAA AAG AAC CCT TGT TAA GGG AGC CAT GTA CCC TGG CAT TG-3') and TQRActin (5'-CTT AAC TCC CTG GCT CAT CAT GGA TCC ACA CGG AGT ACT TGC-3'). The sequence of the ROX-labelled mimic probe used in real-time PCR was 5'-CCG ACA GGA TGC AGA AGG AGA TCA-3'.

The 25-μl PCR mixture comprised 2.5 μl of 10× PCR-buffer II (Applied Biosystems, Foster City, CA, USA), 2.5 mM MgCl_2_, 0.2 mM of each of the four dNTP, 0.15 μM of each of the primer TQF and TQR, 0.25 μl (1.25 U) of AmpliTaq Gold DNA polymerase (Applied Biosystems), and 0.1 μM of each probe. Reaction mixtures were placed in a Rotor-Gene 3000 (Corbett Research, Cambridge, UK) and amplification was performed according to the protocol of Everett and others [29]. The results were analysed with the Rotor-Gene software version 5.0.

The sensitivity of the PCR was estimated to one inclusion forming unit (IFU) per PCR by spiking semen and tissue samples prior to DNA extraction with ten-fold dilutions of *Cp. abortus *(inactivated strain S26/3 in original concentration of 3 × 10^8 ^IFU/ml, kindly provided by D. Longbottom, Moredun Research Institute, UK).

### Detection of antibodies to *Cp. abortus*

For detection of antibodies the Pourquier^® ^ELISA *Chlamydophila abortus *serum verification kit (Montpellier, France) was applied. The ELISA uses a recombinant fragment of an 80-90 kDa polymorphic outer membrane protein and detects antibodies against *Cp. abortus*. The assay was used according to the manufacturer's instruction with S/P% values ≥ than 100 as positive for cattle.

## Results

All 21 semen and 43 cauda epididymidis samples were negative in the PCR. The internal amplification control (mimic) worked well for all samples analysed.

None of the 21 and 43 paired-sera from dairy and beef bulls, respectively, were positive in the antibody detection assay. Most samples were clustered well below the cut-off value 100. Only six samples had S/P% above 20, where 42 was the highest value.

## Discussion

In this study we found no presence of chlamydial agent in any semen or cauda epididymidis tissue samples, i.e. all samples were negative by real-time PCR. This is in concordance with an Austrian study [31] where neither *Cp. abortus *nor *Cp. pecorum *was detected in 273 semen samples from bulls at five AI centres. On the other hand, the results contradict those reported from other investigations performed in apparently healthy bulls. In Lithuania as much as 29.8% of 47 tested bulls had chlamydial agent in their semen, as judged by PCR [13], and chlamydiae were detected by immunofluorescence in 14.3% of 42 bovine ejaculates from the Czech republic [32]. In German and Swiss investigations of semen samples, 9.2% and 6.6%, respectively, were found positive by PCR [22,23].

The sensitivity of the PCR assay was estimated to 1 IFU per PCR with no indication of potential inhibitory factors. In a previous investigation of cows from dairy herds with reproductive disorders we identified positive specimens, including vaginal swabs, placenta and milk when using the same PCR assay [33]. Moreover, several positive specimens from different organs in pigs and placentae in sheep (unpublished data) as well as conjunctival and nasal swabs from cats [34] have been demonstrated by the same PCR at our laboratory. Those samples were handled and stored in a similar way as in the present study. Therefore, the test is considered robust and to have a high sensitivity and specificity.

All sera were negative in the *Cp. abortus *ELISA assay with values far below the cut-off value. The specificity of the test has been reported to be 100% when used to analyse Scottish sheep documented free of *Cp. abortus *[35] and 90% when sera from New Zealand, a country free from *Cp. abortus*, were analysed [36]. The sensitivity were estimated to 91% and 80%, respectively, when analysing sera from experimentally *Cp. abortus *infected sheep [35,36]., and it can, hence, not be excluded that some of our sera were positives but not detected by the test. However, the fact that all the beef bulls, which came from as many as 124 different herds from all over Sweden, were still seronegative after they had been housed together for six months, in adjacent pens under the same roof, indicates that *Cp. abortus *is not present in Swedish beef cattle herds. Moreover, the absence of seropositives among the analysed dairy bulls indicates that *Cp. abortus *is very rare, or absent, in Swedish bulls. These results are in agreement with a previous study in Swedish dairy cows where only 2 out of 525 sera were positive in the same ELISA and only *Cp. pecorum *were confirmed in vaginal swabs [33].

## Conclusions

This study suggest the risk for venereal transmission of chlamydial infection through Swedish bull semen is low. However, because *Chlamydophila *spp. infection rates seem to differ throughout the world, it is essential to clarify the relative importance of transmission of the infection through semen on cattle fertility.

## Competing interests

The authors declare that they have no competing interests.

## Authors' contributions

ACK participated in the design and coordination of the study, drafted and rewrote the manuscript, carried out the PCR and interpreted the results. SE implemented the PCR systems, constructed the mimic and interpreted the results. CB and SA participated in the design and coordination of the study. YP sampled and wrote about the beef bulls. All authors have been involved in revising the manuscript.
